# Current Trends in Neonatal Surgery in India

**Published:** 2012-04-01

**Authors:** Sushmita N Bhatnagar, Yogesh Kumar Sarin

**Affiliations:** Department of Paediatric Surgery, B.J. Wadia Hospital for children, Parel, Mumbai-400 012, India; 1 Department of Pediatric Surgery, Maulana Azad Medical College, New Delhi-110002, India

A new baby is like the beginning of all things-wonder, hope, a dream of possibilities- Eda J. Le Shan

But what about a baby born with congenital anomaly?

Even when antenatal diagnosis of a congenital anomaly is made, there is tremendous psychological impact when such a baby is born. The hopes and dreams of parents are engulfed with fear of death or long-term morbidity. The extent of intervention to correct the anomaly by the treating physician/ neonatologist/ neonatal surgeon could vary depending on where this baby was born. In the resource challenged nations like India, it may also depend on the socio-economic status of the family.

The overall prevalence of congenital anomalies is 3-5%, but may vary from country to country [1]. EUROCAT (European Surveillance of Congenital Anomalies) recorded a prevalence of 2.3.9% during the period 2003-2007. The prevalence rates from India, Taiwan and United Arab Emirates have been reported as 3.6%, 4.3% and 7.9% respectively [2].

Neonatal surgery is a highly specialized and the most sophisticated field of Pediatric Surgery. In the era when organ-specific super-specialties are waging turf wars on pediatric surgery, the latter remains unthreatened at least for neonatal surgery. Neonatal surgery is indeed the jewel in the crown of our specialty. 

Many conditions like Hirschsprung’s disease, hypertrophic pyloric stenosis, etc were recognized as early as 18th century. However, for more than a century, there was no real treatment available; babies were delivered at home and remained untreated or inappropriately treated. The first contribution to the development of neonatal surgery started came from the French midwives and obstetricians and not from the pediatricians, as generally perceived.

The first neonatal surgical unit was opened in Alder Hey Hospital, Liverpool in 1953 by Rickham who has made significant contributions to neonatal surgery [3]. Professor Zachary followed the lead in Sheffield when a separate neonatal surgical ward was opened in early 1964. Many such specialized centers came up all across the world and in 1976, a concept of surgical NICU design according to the level of complexity of care as per the Guidelines was published by the American Academy of Pediatrics [4]. With rising sophistication, progress in neonatal medicine, improving methods of care and support, the survival of the surgical neonates started improving. The forerunners in starting dedicated neonatal surgical intensive care units in India included Wadia Childrens’ Hospital, Mumbai and AIIMS, New Delhi. Presently, India could boast of many similar surgical NICUs, most of them offering level 1 and 2 care. A few surgical NICUs in major metro cities of India are providing level 3 care. In these surgical NICUs, neonatal ventilators (occasionally even high frequency ventilators), phototherapy units, infusion pumps for dose adjusted delivery of intravenous fluids and electrolytes, total and partial parenteral nutrition, broad spectrum antibiotics for control of sepsis and facilities for central line insertions and invasive monitoring and advanced care are available. But all this has not happened overnight in even these centres. It was not too long ago when neonates were admitted and treated in the general pediatric wards, where no neonatal ventilators were available. Special needs in terms of thermoregulation, fluid and electrolyte requirements were not recognized; even proper incubators were not available

The buzz word ‘diversity’ prevails even in neonatal surgery in India. There are places where antenatal diagnosis and termination in fetuses with congenital anomalies is rampant to those where no antenatal diagnosis is made and post-nataly, no attempt is made to treat the baby. Even teaching departments in many state medical colleges lack appropriate facilities and infrastructure and appropriate expertise in this highly sophisticated field of pediatric surgery. More than 50% of newborns probably don’t even reach the hospitals and it is impossible to accurately assess the incidence of the anomalies. What happens to these newborns is obvious. They are either managed by untrained, unskilled professionals with lifelong morbidity or they die a silent death due to lack of medical facilities within reach. More than 50% of the infant mortality is during the neonatal period [5], with congenital anomalies being a major contributory factor. It is obvious that we still have a long way to go for developing this subspecialty in our country.

The exact status of neonatal surgery in India has been scantly documented [6,7]. Gupta et al [8] had concluded a decade ago that the results of surgery for the same condition were very variable in different centers. The reasons for poor survival were diverse, but it was noticed that the amount of work load inversely correlated with the results. In spite of sophistication in technology, they observed that the overall survival rates in India were far inferior to that noted in the centers from the developed world.

The authors undertook a fresh national pilot survey of the existent neonatal surgical facilities. A short questionnaire was sent to 12 centers in different parts of the country; consultants from 7 centers from the major cities of India such as Mumbai, New Delhi, Lucknow, Bangalore and Chennai responded. The number of neonatal surgeries performed annually in these centers ranged from 60 to 351. Ano-rectal malformation, esophageal atresia and/ or tracheo-esophageal fistula, intestinal atresias, Hirschsprung’s disease, and neural tube defects were the commonest anomalies that are dealt in the neonatal age. The center in Chennai observed an increased rate of congenital diaphragmatic hernia and lung lesions, whereas the center in Lucknow had increased rate of abdominal wall defects such as exomphalos and gastroschisis. Except for 3 centers, most of the Institutes had surgical beds in the neonatal medical intensive care unit, and majority of them were providing joint care to the surgical neonates, which included pre and post-operative care as well as ventilatory management. The mortality rate for the surgical neonates ranged from 5% to 16%, and sepsis (as high as 80%) was the major contributor to mortality in all the centers. We intend to send the questionnaire to 1500 –odd pediatric surgeons working in SAARC countries to get more meaningful data.

The outcome of conditions esophageal atresia has improved tremendously from 0% to almost 95-100% in some centers over the years (unpublished data), but there are several major issues such as sepsis leading to increased overall mortality.

The way forward in India and the other developing countries is:

1. To create a national neonatal registry.
2. To develop awareness amongst healthcare professionals and pediatric surgeons regarding the outcome of congenital anomalies when treated by a trained neonatal surgeon.
3. To redesign the surgical NICU facilities and as per the American Academy guidelines of Level 1, Level 2 and Level 3 care.
4. To create more tertiary care neonatal surgical intensive care units so that high standard care can be given to all the neonates.
5. To improve the rate of neonates reaching the tertiary treatment centers for management of anomalies.
6. As per the results of our pilot survey, sepsis seems to be the major killer of surgical neonates. A detailed survey is proposed to assess the incidence and causation of sepsis in the surgical neonates. Proactive measures to reduce the rate of sepsis are of paramount significance to reduce the neonatal mortality rate.

## Footnotes

**Source of Support:** Nil

**Conflict of Interest:** None declared

